# Induction of Cytokines by Nucleic Acid Nanoparticles (NANPs) Depends on the Type of Delivery Carrier

**DOI:** 10.3390/molecules26030652

**Published:** 2021-01-27

**Authors:** Yelixza I. Avila, Morgan Chandler, Edward Cedrone, Hannah S. Newton, Melina Richardson, Jie Xu, Jeffrey D. Clogston, Neill J. Liptrott, Kirill A. Afonin, Marina A. Dobrovolskaia

**Affiliations:** 1 Nanoscale Science Program, Department of Chemistry, University of North Carolina Charlotte, Charlotte, NC 28223-0001, USA; yavila@uncc.edu (Y.I.A.); mchand11@uncc.edu (M.C.); mricha91@uncc.edu (M.R.); 2 Nanotechnology Characterization Laboratory, Cancer Research Technology Program, Frederick National Laboratory for Cancer Research Sponsored by the National Cancer Institute, Frederick, MD 21702, USA; edward.cedrone@nih.gov (E.C.); hannah.newton@nih.gov (H.S.N.); jie.xu@nih.gov (J.X.); clogstonj@mail.nih.gov (J.D.C.); 3 Department of Pharmacology and Therapeutics, Institute of Systems, Molecular and Integrative Biology, University of Liverpool, Liverpool L7 3NY, UK; neill.liptrott@liverpool.ac.uk

**Keywords:** nucleic acid nanoparticles, NANPs, cytokines, vaccines, immunotherapies, immunology

## Abstract

Recent insights into the immunostimulatory properties of nucleic acid nanoparticles (NANPs) have demonstrated that variations in the shape, size, and composition lead to distinct patterns in their immunostimulatory properties. While most of these studies have used a single lipid-based carrier to allow for NANPs’ intracellular delivery, it is now apparent that the platform for delivery, which has historically been a hurdle for therapeutic nucleic acids, is an additional means to tailoring NANP immunorecognition. Here, the use of dendrimers for the delivery of NANPs is compared to the lipid-based platform and the differences in resulting cytokine induction are presented.

## 1. Introduction

The field of RNA and DNA nanotechnology is rapidly growing. In the past decade, researchers have established various approaches to synthesize RNA and DNA nanoassemblies of different sizes, shapes, and compositions and generated proof-of-concept data intended for the use of these materials in biology and medicine [1,2,3,4,5,6,7,8,9,10,11]. A growing library of nucleic acid nanoparticles (NANPs), the design of which takes advantage of natural RNA (and DNA) motifs and canonical Watson–Crick base pairings, have been demonstrated to assemble into precise nanoscaffolds exemplified by hexagonal rings [12], various polygons [13], and fibrous structures [14], to name a few [15]. A variety of NANPs are now being investigated for broad applications in detection and diagnostics [16,17,18], targeting specific disease sites [19], and as therapeutic approaches [9,20,21,22] for various illnesses. As the technology approaches the stage of preclinical development and clinical translations, many researchers in the field have consolidated their efforts to overcome translational gaps and accelerate the transition of DNA and RNA nanoassemblies from bench to clinic [23,24,25,26,27,28]. Among these efforts is the understanding of the immunological properties of NANPs as a new class of therapeutic nucleic acids.

Our group has recently reported that biomarkers for NANP immunorecognition are type I and type III interferons (IFNs), which are produced by human primary blood cells only after NANPs are delivered with a widely used lipid-based carrier (Lipofectamine 2000 or L2K); otherwise, without a delivery agent, NANPs are not efficiently internalized and do not induce an IFN response [29]. Among other structure–activity relationships, we demonstrated that the IFN-inducing capability of NANPs depends on their composition (RNA-based NANPs are more potent than their DNA counterparts), shape (globular structures are more potent than planar particles, which in turn are more potent than fibrous NANPs), and size [29]. This relationship is well-exemplified by DNA and RNA cubes, which are both six-stranded 3D NANPs similar in size, shape, and sequence. While both DNA and RNA cubes have been demonstrated to serve as nanoscaffolds for carrying therapeutic nucleic acids into cells, the difference in their DNA versus RNA composition has been shown to yield greater IFN induction for RNA cubes when compared to their DNA analogs [30]. The most remarkable finding of our earlier studies was that despite general knowledge regarding the involvement of toll-like receptors (TLRs) in the recognition of DNA and RNA, TLR7, known as a receptor for single-stranded RNA, played a key role in the immune recognition of both DNA and RNA cubes [29,31]. Altogether, the results of our studies allowed us to hypothesize that both the quality (i.e., the repertoire of cytokines) and quantity (i.e., the magnitude of the cytokine response) of the immune response to NANPs can be manipulated not only by changing NANPs’ physicochemical properties and composition, but also by using different types of carriers [29,31].

As a candidate for such a delivery platform, polyamidoamine (PAMAM) dendrimers are cationic, hyperbranched, globular structures. Amine-terminated PAMAM dendrimers, like the ones used in this study, have been proposed as an effective delivery platform for gene therapy by complexation with siRNAs, biological molecules, and drugs [32,33,34,35,36,37]. Different generations of the PAMAM dendrimers have been shown to successfully carry nucleic acids such as plasmids, siRNAs, and miRNAs into different cancer cell lines [38,39,40]. Once inside the cells, the siRNAs were able to activate RNA interference and silence their specific target mRNAs in both in vitro and in vivo proof-of-concept models [41]. The dendrimer–nucleic acid complexes form through electrostatic interactions between the positively charged amine group terminals of the dendrimers and the negatively charged phosphate groups of the nucleic acids [42]. 

Herein, we present the results confirming the hypothesis that immunostimulation by NANPs can also be manipulated by the type of carrier. Specifically, we compared the cytokine induction by DNA and RNA cubes delivered to human peripheral blood mononuclear cells (PBMCs) using either L2K (the carrier used in our previous studies) or generation 5 amine-terminated (G5-NH_2_) polyamidoamine (PAMAM) dendrimers. The results of DNA and RNA cubes’ physicochemical characterization, complexation with G5-NH_2_ PAMAM dendrimers, resistance to nucleases, and delivery to cancer cells and PBMCs are also presented. 

## 2. Results

### 2.1. Physicochemical Characterization of Dendrimers 

**Figure 1:**
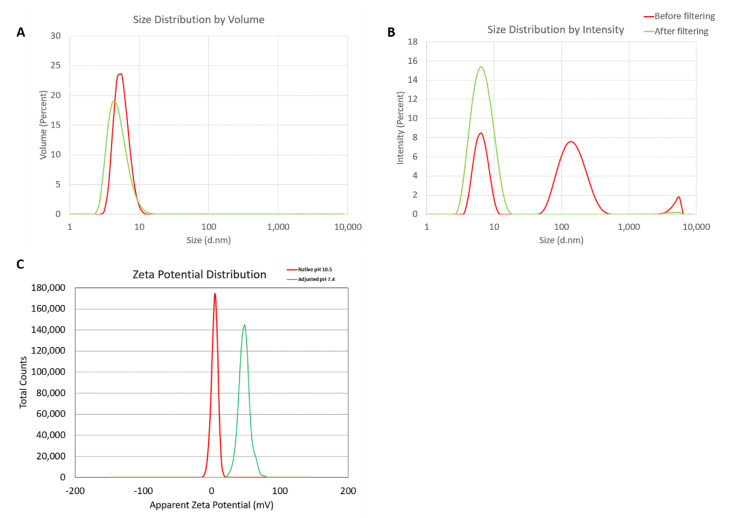
Physicochemical characterization of the G5-NH_2_ PAMAM dendrimers. The averaged intensity (**A**) and volume (**B**) distribution plots as measured by dynamic light scattering and the averaged zeta potential distribution (**C**). The hydrodynamic size was measured before and after filtration through a 0.02 µm filter. Zeta potential was measured both at its native pH and after adjustment to neutrality.

Hydrodynamic sizes were measured by dynamic light scattering (DLS) for the dendrimer as is (no filtering) and after filtration through a 0.02 µm filter. The intensity, volume distribution, and zeta potential plots are shown in Figure 1 and summarized in Table 1. Before filtration, several peaks are observed in the intensity-weighted distribution plot (Figure 1A), with the most dominant size population being ~7 nm, as determined by the volume-weighted distribution plot (Figure 1B). After filtration, these larger size populations (consisting of aggregates) are removed and a monomodal size distribution centered at 7 nm (Int-Peak) is observed. 

The zeta potential distributions for the dendrimer are shown in Figure 1C and summarized in Table 2. Zeta potential was measured both at its native pH and after adjustment to neutrality (Figure 1C). At its native pH (10.5), the dendrimer is neutral (+4.6 mV) due to the surface primary amines existing as NH_2_. Note, zeta potentials from –10 to +10 mV are generally considered neutral. The zeta potential becomes highly cationic (+48.2 mV) after pH adjustment to 7.4 as the surface primary amines are protonated and exist as NH_3_^+^.

### 2.2. NANP Synthesis and Characterization

**Figure 2:**
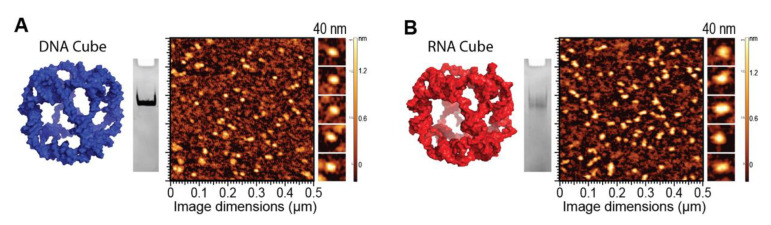
DNA and RNA cube characterization. 3D models, native-PAGE results, and representative AFM images of (**A**) DNA cubes and (**B**) RNA cubes.

To demonstrate the ability of G5-NH_2_ dendrimers to serve as a carrier of NANPs, representative DNA and RNA cubic NANPs were chosen as a proof of concept for all experiments. These NANPs have been previously characterized and have been demonstrated to be delivered into cells using a variety of delivery platforms. While both exhibit the same globular shape and relative size, their difference in composition in terms of being made of either DNA or RNA makes for a noticeable divergence in their immunostimulation, with RNA cubes serving as potent stimulators of IFNs. DNA and RNA cubes were assembled in endotoxin-free conditions and were visualized via non-denaturing polyacrylamide gel electrophoresis (native-PAGE) to verify their assembly and additionally visualized via atomic force microscopy (AFM) to ensure sample uniformity (Figure 2).

### 2.3. NANP Complexation with G5-NH_2_ Dendrimers

The electrostatically-driven complexation of G5-NH_2_ dendrimers to NANPs was assessed using the number of primary amines available per dendrimer (N) and the number of phosphates available on the backbone of a DNA duplex (P) to calculate complexation at the N/P ratio. Once DNA duplexes were complexed to G5-NH_2_ dendrimers at different N/P ratios and incubated for 30 min, the samples were visualized via agarose gel electrophoresis (supporting Figure S1) to determine the ratio at which the DNA duplex migration was impeded. This ratio was then used to determine the amounts of G5-NH_2_ needed to bind NANPs. 

**Figure 3:**
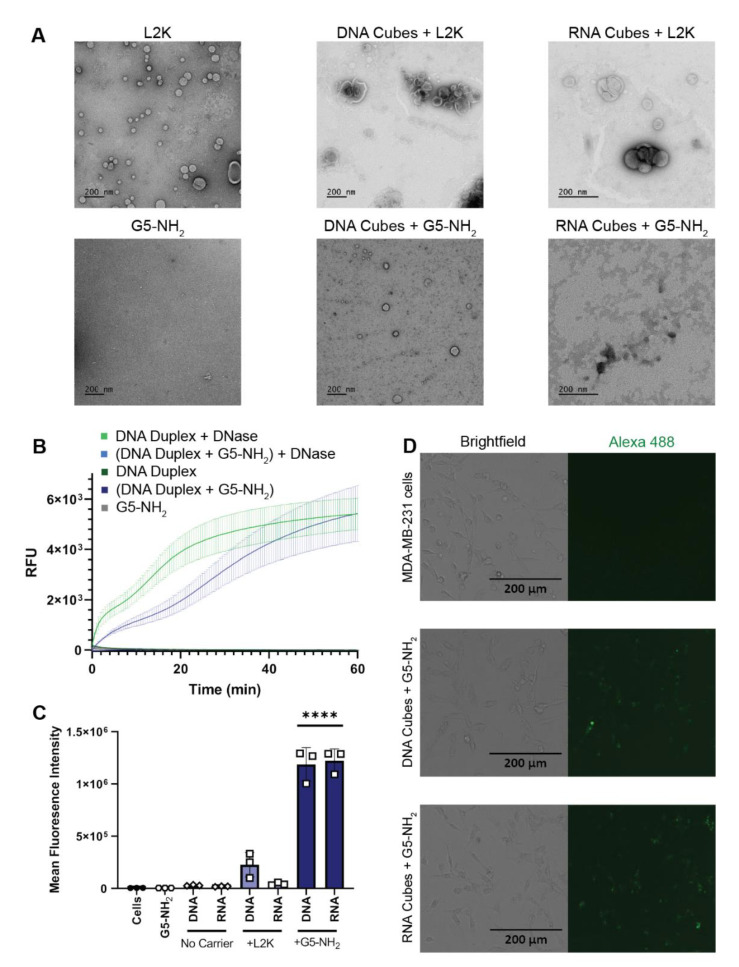
NANPs complexed with G5-NH_2_ dendrimers. (**A**) Transmission electron microscopy images of Lipofectamine 2000 (L2K), G5-NH_2_ dendrimers, and DNA and RNA cubes complexed to either L2K or G5-NH_2_ dendrimers. (**B**) Resulting fluorescence profiles from nuclease resistance assays. (**C**) Mean fluorescence intensity associated with the in vitro uptake of Alexa 488-labeled DNA and RNA cubes in MDA-MB-231 cells. Each bar shows the mean response and standard deviation (*N* = 3). Statistical significance between the DNA and RNA cubes delivered with G5-NH_2_ versus all other treatments is denoted by **** where *p* < 0.0001. (**D**) Brightfield and GFP microscopy images of MDA-MB-231 cells transfected with DNA cubes complexed to G5-NH_2_ dendrimers and RNA cubes complexed to G5-NH_2_ dendrimers.

L2K and G5-NH_2_ dendrimers were visualized individually with transmission electron microscopy (TEM) and then again with the addition of cubic NANPs (Figure 3A). To investigate whether NANPs could be complexed to and protected by the G5-NH_2_ dendrimers, a nuclease resistance assay was conducted. To run this assay, a DNA duplex, decorated with a fluorophore/quencher pair, was complexed to G5-NH_2_ dendrimers and treated with DNase. The change in fluorescence over time for the G5-NH_2_-complexed dendrimers was compared to uncomplexed duplexes (Figure 3B). Contrarily to the uncomplexed duplexes, G5-NH_2_-complexed duplexes were protected from nuclease digestion for an extended period of time (one hour). The delay in fluorescence increase of the G5-NH_2_-complexed duplexes indicated that the dendrimers protected the duplexes from nuclease degradation, thus again confirming the complexation between nucleic acid constructs and dendrimers. 

To evaluate DNA and RNA cubes’ uptake efficiency by a cancer cell line when complexed to either dendrimers or L2K, Alexa 488-labeled cubes were used to track the complexes introduced into the human breast cancer cell line MDA-MB-231 (Figure 3C,D). The uptake results provided information on the overall general uptake of the G5-NH_2_ cubes in an adherent cell line that is customarily used to assess NANP uptake with other carriers. The cells appeared to uptake the G5-NH_2_-complexed cubic NANPs significantly more than those observed for the L2K-complexed NANPs. Uptake of the complexes was observed through the increase in mean fluorescence intensity of the treated cells. 

### 2.4. Cytokine Response in PBMCs Depends on the Type of Carrier and Correlates with NANP Uptake by the Cells

**Figure 4:**
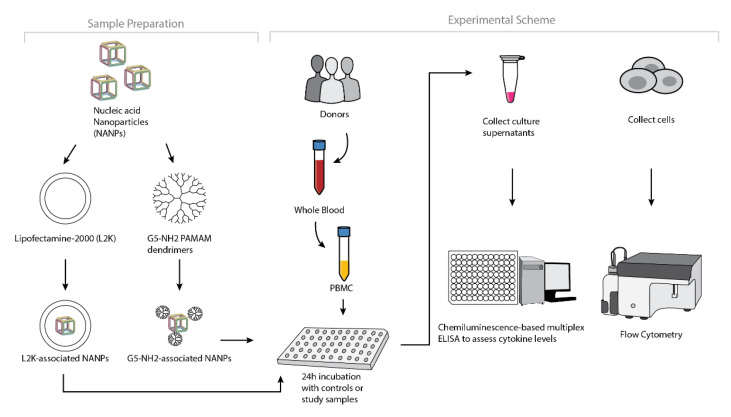
Experimental flow of the complexation of DNA and RNA cubes with either G5-NH_2_ dendrimers or L2K and their further analysis in PBMCs.

**Figure 5:**
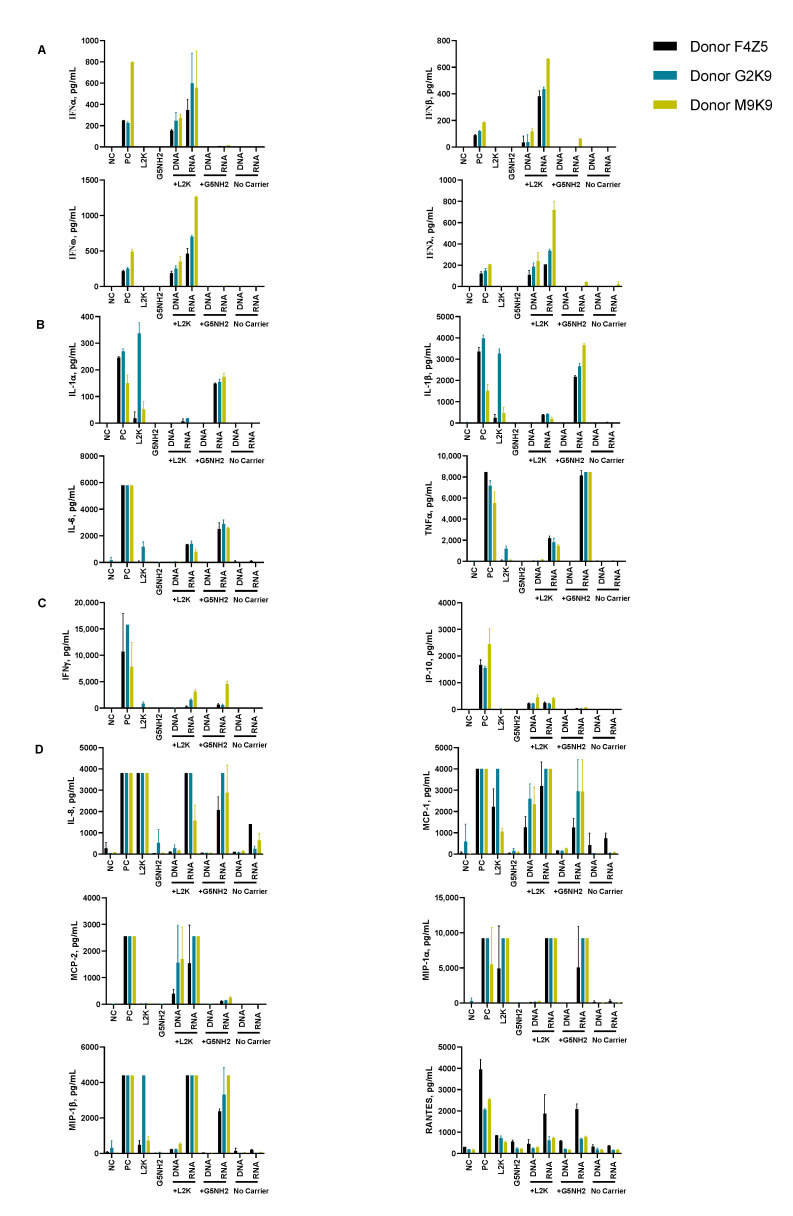
Cytokine induction by DNA and RNA cubes as a function of the delivery carrier. PBMC from three healthy human donor volunteers (F4Z5, G2K9, and M9K9) were treated with negative control (NC), positive control (PC), DNA cubes, or RNA cubes for 24 h. Prior to the addition to PBMC cultures, DNA cubes and RNA cubes were complexed with lipofectamine 2000 (L2K), G5 amine-terminated PAMAM dendrimers (G5-NH_2_) or used without complexation (no carrier). Culture supernatants were analyzed for the presence of cytokines, chemokines, and interferons using multiplex ELISA as described in the Materials and Methods. The data are presented based on the function of cytokines, including (**A**) type I and type III interferons, (**B**) danger signals and cytokines commonly associated with trauma and cytokine storm, (**C**) type II interferon and type II interferon-inducible protein, and (**D**) chemokines. Each bar shows the mean response and standard deviation (*N* = 2). Other cytokines from this study are presented on Figure S3.

To understand whether the spectrum and the magnitude of the cytokine response to DNA and RNA cubes depend on the type of carrier, we conducted experiments using human PBMCs (Figure 4). NANPs were added to PBMC cultures either without a carrier or after complexation with either L2K or G5-NH_2_ dendrimers, and the supernatants were analyzed for the presence of 29 cytokines. Owing to the pleiotropic function of cytokines, we used the broadest panel available; and for the purpose of this manuscript, when analyzing the results, we grouped cytokines based on their known roles in various biological responses as will be detailed below. Analysis of culture supernatants revealed that NANPs used without a carrier and G5-NH_2_ dendrimers alone did not induce any cytokines (Figure 5 and Figure S3).

After the complexation with L2K, both DNA and RNA cubes induced type I and type III interferons, known for their role in anti-viral and anti-tumor effects; these responses were stronger in the RNA cube-treated group than in the DNA cube-treated group (Figure 5A). Unlike L2K-complexed NANPs, particles complexed with amine-terminated dendrimers did not induce type I and type III IFNs (Figure 5A). 

A striking difference, however, was observed for cytokines that are known as danger signals (IL-1α) and those commonly associated with stress, trauma, and cytokine storm (IL-1 β, IL-6, TNFα). In this case, L2K-complexed NANPs did not produce a response, whereas dendrimer-delivered NANPs induced the aforementioned stress and danger-related cytokine biomarkers (Figure 5B). Similar to the effect on type I and type III IFNs observed in the L2K-delivered NANPs, RNA cubes delivered using dendrimers were more potent in inducing stress-related cytokines than DNA cubes; no cytokines were detected in the samples treated with DNA or RNA cubes without a carrier (Figure 5A,B). Interestingly, L2K alone induced IL-1α and IL-1β and, in PBMCs from one donor, low levels of TNFα and IL-6; however, this effect was neutralized by the complexation with RNA and DNA cubes (Figure 5B). 

Low levels of type II interferon (IFNγ), known for its role in T cell-mediated immunity, were observed in the L2K-delivered NANP group and similar between DNA cubes and RNA cubes (Figure 5C). IFNγ-induced protein (IP-10), however, was detected only in the L2K-delivered NANP group (Figure 5C). Similar to the data with other cytokines, DNA and RNA cubes used without a carrier did not induce type II IFN and IFNγ-induced protein (Figure 5C).

Analysis of chemokines (IL-8, MIP-1α, MIP-1β, MCP-1, MCP-2, and RANTES) revealed that L2K alone induced all chemokines except for MCP-2, and this effect was neutralized by complexation with NANPs; dendrimers alone did not induce any of these chemokines (Figure 5D). Interestingly, induction of IL-8, MIP-1α, MCP-1, and RANTES was similar between L2K- and dendrimer-delivered NANPs and was stronger in RNA cubes than in DNA cubes (Figure 5D). In contrast, the induction of MCP-2 was observed only in L2K-complexed NANPs, but not in dendrimer-complexed NANPs and was again higher with RNA cubes than with DNA cubes (Figure 5D). The pattern of MCP-2 induction (Figure 5D) matched closely with that of type I and type III IFNs (Figure 5A). Other cytokines (IL-2, IL4, IL-5, IL-22, IL-10, IL-12, and IL-21) were also detected; the induction of some of these biomarkers (e.g., IL-2 and IL-15) was donor-dependent (Figure S3).

**Figure 6:**
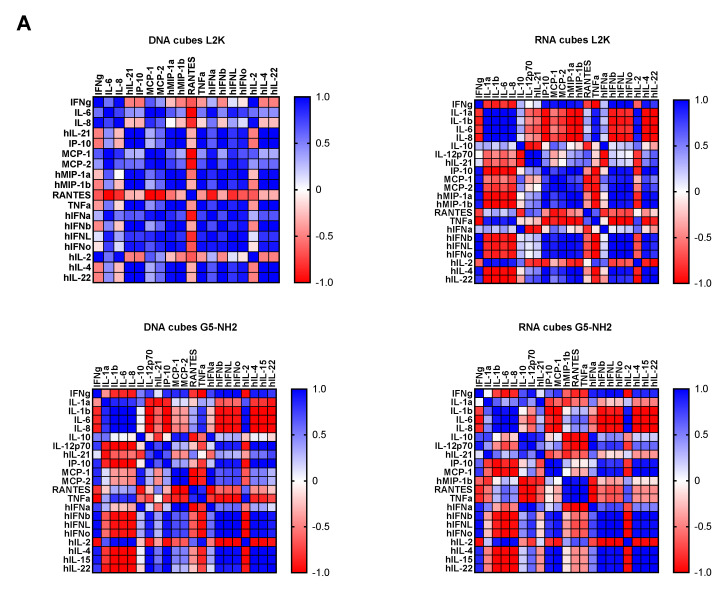

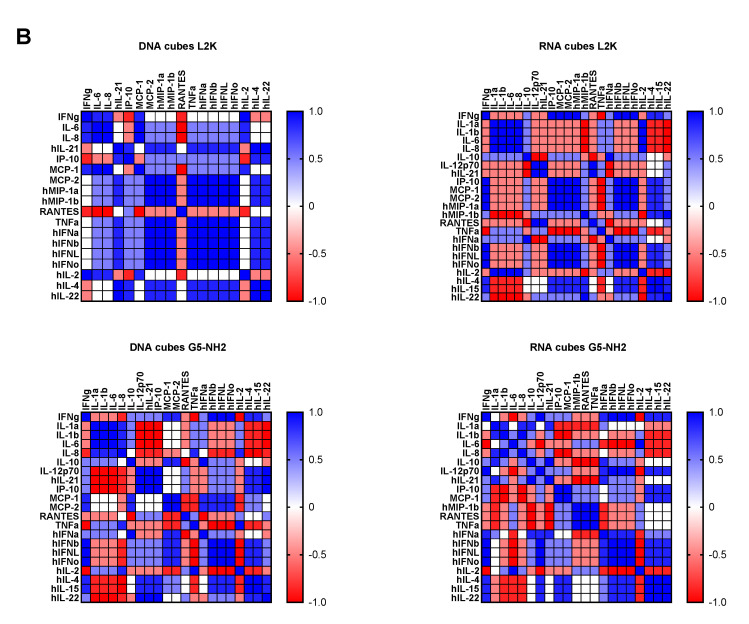
Correlation analysis of cytokine response. The data from the multiplex cytokine analysis including those presented in Figure 5 were analyzed using the GraphPad Prism software to determine a correlation or lack thereof between individual cytokines. (**A**) The Pearson correlation matrix assumes a Gaussian distribution (parametric analysis). In this analysis, values between ±0.5 and ±1 refer to a high degree of correlation, whereas values close to ±1 mean perfect correlation; negative values (in red) refer to the negative correlation, whereas positive values (in blue) mean positive correlation. (**B**) The Spearman correlation matrix assumes no Gaussian distribution (non-parametric analysis). In this analysis, values of ±1 mean perfect correlation; the closer the value is to zero, the weaker the association is; negative values (in red) and positive values (in blue) refer to the negative and positive correlation, respectively.

To understand whether detected cytokines provide positive or negative regulation loops that influence their expression, we conducted a correlation analysis. Since the number of donors was limited, we applied two matrices—the Pearson matrix, which assumes a Gaussian distribution (Figure 6A), and the Spearman matrix, which assumes a non-Gaussian distribution (Figure 6B). With L2K-delivered DNA cubes, a positive correlation was observed between type II IFN (IFNγ) and cytokines and chemokines IL-6, IL-8, MCP-1, MCP-2, and IL-2, and between type I and type III IFNs (IFNα, IFNβ, IFNω, IFNλ) and cytokines and chemokines MCP-1, MCP-2, TNFα, IL-4, and IL-22 (Figure 6A, DNA cubes-L2K). A negative correlation in the same group was detected between type I and type III IFNs and chemokine RANTES (Figure 6A, DNA cubes-L2K). With L2K-delivered RNA cubes, a positive correlation was observed between individual cytokines IL-1α, IL-1β, IL-6, IL-8, and IL-2; IFNγ and chemokines MCP-1, MCP-2, MIP-1α, and MIP-1β; type I and type III IFNs; chemokines MCP-1, MCP-2, MIP-1α, and MIP-1β and type I and type III IFNs; and between IL-4 and IL-22 (Figure 6A, RNA cubes L2K). A negative correlation was observed between cytokines IL-1α, IL-1β, IL-6, and IL-8 and all IFNs, IL-4, and IL-22 (Figure 6A, RNA cubes-L2K). With dendrimer-delivered DNA cubes, a positive correlation was observed between individual cytokines IL-1α, IL-1β, IL-6, and IL-8; between individual type I and type III IFNs; IL-4, IL-15, and IL-22; and between IFNγ and MCP-1, MCP-2, type I and type III IFNs, IL-4, IL-15, IL-22, IL-12, and IP-10 (Figure 6A, DNA cubes-G5-NH_2_). Negative correlation in the same treatment group was observed between IL-1α, IL-1β, IL-6, IL-8, and all IFNs, IL-12, IL-21, IP-10, IL-4, IL-15, IL-22, MCP-1, MCP-2, and RANTES; and between individual cytokines IL-4, IL-15, and IL-22 (Figure 6A, DNA cubes-G5-NH_2_). With dendrimer-delivered RNA cubes, a positive correlation was observed between IFNγ and IL-12, MCP-1, type I and type III IFNs, IL-4, IL-14, and IL-22; between IL-6, IL-1α, IL-1β, and IL-8; MIP-1α, MIP-1β, RANTES, and TNFα; between type I and type III IFNs and IL-4, IL-15, and IL-22; and between individual type I and type III IFNs (Figure 6A, RNA cubes-G5-NH_2_). Negative correlation in this treatment group was observed between IL-1α, IL-1β, IL-6, and IL-8, and MIP-1α, MIP-1β, IL-4, IL-15, and IL-22; between TNFα and IFNγ, IP-10, MCP-1, MCP-2, MIP-1α, MIP-1β, and type I and type III IFNs; between IL-2 and IL-4, IL-15, and IL-22 (Figure 6A, RNA cubes-G5-NH_2_). While the correlation indices for individual cytokines were different in the Spearman matrix, the overall conclusions about negative and positive correlation did not change (Figure 6B).

**Figure 7:**
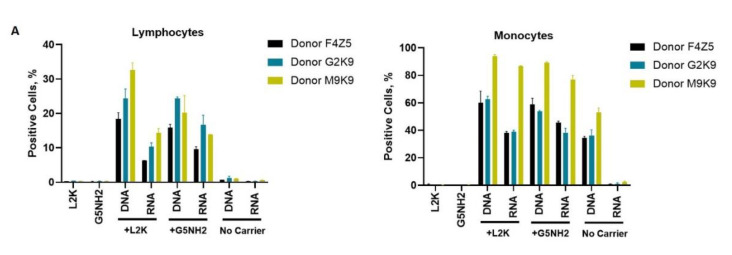

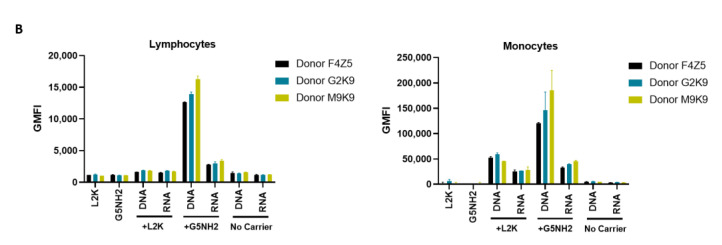
Uptake of fluorescently labeled DNA and RNA cubes by peripheral blood cells. PBMCs from three healthy human donor volunteers (F4Z5, G2K9, and M9K9) were either left untreated or incubated with Alexa 488-labeled DNA cubes or RNA cubes for 24 h. Prior to the addition to PBMC cultures, DNA cubes and RNA cubes were complexed with Lipofectamine 2000 (L2K), G5 amine-terminated PAMAM dendrimers (G5-NH_2_) or used without complexation (no carrier). After a wash to remove excess particles, the cells were analyzed by flow cytometry as described in the Materials and Methods. (**A**) Analysis of the percentage of positive cells indicates the overall proportion of the cells in either the lymphocyte or monocyte population associated with the fluorescent signal that is greater than that in the carrier alone or untreated cells. (**B**) Analysis of geometric mean fluorescent intensity (GMFI) reveals the degree of a fluorescent signal associated with the individual cells in the lymphocyte or monocyte populations. Green fluorescence is delivered to the cells by DNA and RNA oligonucleotides labeled with Alexa 488 prior to their assembly into DNA and RNA cubes, respectively. Each bar shows the mean response and standard deviation (*N* = 2).

Next, we tested NANP uptake by blood cells. PBMCs from the same donors as those used for cytokine analysis were exposed to carriers alone (L2K or G5-NH_2_ dendrimers), DNA cubes or RNA cubes without a carrier, or DNA cubes or RNA cubes complexed with either L2K or with dendrimers (Figure 7). The NANPs used in this study contained a green fluorescent label (Alexa 488) covalently attached to one oligonucleotide of each six-stranded assembly of the DNA and RNA cubes. Percent of positive cells shows the proportions of cells in the analyzed population of lymphocytes or monocytes that were associated with green fluorescence, which, in turn, is indicative of the particle uptake and/or association with the cellular membrane. When L2K or dendrimers were used as delivery carriers for NANPs, between 40 and 90% of monocytes demonstrated a greater fluorescent signal as opposed to 10–30% of lymphocytes (Figure 7A). The uptake of NANPs in L2K and dendrimer-complexed groups was comparable in both monocytes and lymphocytes; a greater uptake of DNA cubes vs. RNA cubes complexed with L2K was noticed (Figure 7A, lymphocytes). No uptake of RNA and DNA cubes delivered without a carrier was seen in lymphocytes and monocytes treated with RNA cubes, while about 40% of the monocytes exposed to DNA cubes without a carrier demonstrated green fluorescence (Figure 7A, monocytes).

When geometric mean fluorescent intensity (GMFI), indicative of the magnitude of NANP uptake by individual cells, was measured, no significant uptake of naked RNA and DNA cubes was noticed in either lymphocytes and monocytes (Figure 7B). No or very low levels of uptake were registered for both DNA and RNA cubes complexed with L2K in lymphocytes (Figure 7B, lymphocytes). The lymphocyte uptake of RNA and DNA cubes complexed with dendrimers was greater than that after the complexation with L2K, and the uptake of DNA cubes complexed with dendrimers was greater than that of the RNA cubes delivered using dendrimers (Figure 7B, lymphocytes). The uptake of both DNA and RNA cubes by monocytes was also greater in the dendrimer group than in the L2K group; in both groups, the uptake of DNA cubes was higher than that of the RNA cubes (Figure 7B, monocytes). In all groups where the uptake was registered, the signal was an order of magnitude higher in monocytes than in lymphocytes (Figure 7B).

## 3. Discussion

Physicochemical properties of PAMAM dendrimers used in this study were consistent with those described earlier [43,44,45]. The DNA and RNA cubes are complexed to G5-NH_2_ dendrimers through electrostatic interactions between the negatively charged phosphate groups of the nucleic acid cubes and the positively charged amine surface groups from the dendrimers. The N/P ratio of cubes to G5-NH_2_ dendrimers was determined by using a gel retardation assay showing that complete binding occurs at a 1.5 N / 1 P ratio. This was shown by the neutralization of the nucleic acids on the gel through the decrease in migration along the gel. A nuclease resistance assay was also used to determine the ability of dendrimers to protect NANPs from nuclease degradation. The results in Figure 3B show that when DNA and RNA cubes are complexed to G5-NH_2_ dendrimers, the rate of digestion by nucleases is lowered and prolonged for up to 60 min. 

The observed induction of type I (IFNα and IFNβ) and type III IFNs (IFNλ) by DNA and RNA cubes complexed with L2K but not by those used without any carrier, the higher potency of IFN induction by RNA cubes vs. DNA cubes (Figure 5A), and the correlation with the uptake by monocytes (Figure 7) are in agreement with our earlier studies [29,31]. Since type I IFNs’ main function is to prevent viral replication in cells and that of type III IFNs is to support anticancer immunity, the data point to the potential utility of L2K-mediated delivery of NANPs in stimulating anti-viral and anti-tumor immune responses. Our hypothesis that by changing the carrier used to deliver NANPs to the blood cells one may control the spectrum and the magnitude of the cytokine responses was verified in the present study. The induction of type I and type III IFNs and proinflammatory cytokines associated with stress and damage are in direct contrast between NANPs delivered using L2K and those complexed with dendrimers (compare DNA cubes and RNA cubes complexed with L2K to those complexed with dendrimers in Figure 5A,B). Cationic dendrimers were shown in multiple studies to affect the integrity of cellular membranes [43,44,45,46,47,48,49]. We also reported earlier that many nanoparticles are immunomodulatory in that a combination of otherwise non-reactive particles produces a detectable biological response [50,51,52]. Our results, therefore, suggest that NANPs delivered by cationic dendrimers are perceived by immune cells as danger signals, hence the induction of IL-1α, IL-6, and IL-8 [24]. Cationic nanoparticles activate the inflammasome, thereby contributing to the secretion of mature IL-1β, expression of which is induced by other stimuli [53,54,55]. The induction of IL-1β observed in supernatants from cells treated with NANP–dendrimer complexes is consistent with this knowledge; the data suggest that NANPs induce IL-1β expression whereas cationic dendrimers activate the inflammasome to produce mature IL-1β proteins. The induction of type II IFN (IFNγ) by L2K- and dendrimer-complexed NANPs (Figure 5C) is new data; to our knowledge, this phenomenon has not been previously reported. IFNγ is produced by activated T cells and its main function is to activate macrophages and various other cell types and to coordinate a cooperation between activated T cells and other host cells. Therefore, these data point to the potential utility of NANPs for controlling adaptive immunity. The induction of chemokines by L2K alone (Figure 5D) is not unexpected; we reported earlier that lipid-based nanoparticles commonly induce chemokines via a mechanism involving oxidative stress [50,51,52,56]. While this induction complicates the interpretation of chemokine results in the NANPs-L2K group, the data suggest that complexation with DNA and RNA cubes neutralizes this effect (Figure 5D), which is consistent with the expected change in the L2K’s overall charge after its electrostatic complexation with NANPs. The induction of other cytokines (IL-2, IL-4, IL-15, IL-22, IL-10, IL-12, and IL-21) was also observed (Figure S3); in some cases (e.g., IL-2 and IL-15), the induction was donor-dependent suggesting that individual variability in NANP-mediated cytokine signaling including the expression of receptors involved in NANP recognition exists. Such interindividual variability is not surprising since both qualitative and quantitative variations in individuals’ immune responses have been described before [57,58,59,60,61]. 

Correlation analysis revealed the complexity of the cytokine network in that both positive and negative correlation was observed between type I, type II, and type III IFNs, chemokines, and various interleukins and TNFα (Figure 6). These observations are consistent with the current knowledge of the cytokines’ pleiotropic function and their ability to regulate their expression via both homo- and hetero-stimulatory mechanisms [62,63]. Cytokine-mediated refractory states have also been reported [64], and it is possible that NANP delivery using different carriers can induce different refractory states, and NANPs’ physicochemical properties can further contribute to these effects. It is important to note that the correlation analysis reveals the strength of the relationship between individual cytokines and is helpful in guiding the mechanistic studies; it is not meant to analyze a quantitative difference between study samples. Furthermore, due to the limited number of donors used in our study, the current correlation analysis should be considered preliminary and used to generate ideas for subsequent mechanistic studies involving PBMC from a greater number of donors. 

These data also point to communication between different cell types such as monocytes (the main producers of TNFα, IL-1, IL-6, IL-8, and MIP-1), plasmacytoid dendritic cells (the main source of type I and type III IFNs), and T lymphocytes (the main producers of chemokines MCP and RANTES, type II IFN, and IL-2). Most importantly, the negative and positive correlation patterns differ between DNA and RNA cubes and between L2K- and dendrimer-delivered NANPs. These data further support the original hypothesis about the NANPs’ ability to stimulate immune responses that might differ both quantitatively and qualitatively depending on the type of carrier used to deliver these particles to the immune cells. It would be interesting to compare routes of uptake and molecular pathways induced by the same types of NANPs after complexation with different carriers; this is the focus of the future research in this field.

The cytokine data (Figure 5 and Figure 6) correlate with NANP uptake by immune cells, which was studied by flow cytometry (Figure 7). The greater rates of NANP uptake by monocytes are consistent with the well-known phagocytic function of these cells [65]. The uptake of naked NANPs is negligible, which explains the lack of cytokine induction by RNA and DNA cubes used without a carrier. Since the melting temperature of DNA cubes is about 37 °C, an increase in the percentage of positive monocytes observed after the exposure to naked DNA cubes is likely due to the disassembly of these particles in the culture medium followed by an interaction of individual DNA oligonucleotides with cells.

## 4. Materials and Methods

### 4.1. Materials

Generation 5 amine-terminated PAMAM dendrimers were purchased from Dendritech (Midland, MI, USA). Lipofectamine 2000 and all cell culture reagents were from Invitrogen (Carlsbad, CA, USA). Reagents for the preparation of buffers were purchased from Sigma-Aldrich (St. Louis, MO, USA).

### 4.2. Physicocheimcal Characterization of Dendrimers 

A Malvern Zetasizer Nano ZS instrument (Southborough, MA, USA) with a back-scattering detector (173°) was used for measuring the hydrodynamic size (diameter) in the batch mode. NIST-NCL joint protocol PCC-1 was followed (https://ncl.cancer.gov/resources/assay-cascade-protocols). Samples were prepared at a concentration of 3 mg/mL in 10 mM NaCl. Samples were measured as is (no filtering) and after filtration through a 0.02 µm filter. Samples were measured at 25 °C in a quartz microcuvette. Traces in the figures represent the average of ten measurements. Hydrodynamic diameters are reported as the intensity-weighted average and as the volume-weighted average over a particular range of size populations corresponding to the most prominent peak. The Int-Peak value is used as the hydrodynamic diameter of a particular species. The Vol-Peak and %Vol values are used to approximate relative amounts of various species in the formulation. A Malvern Zetasizer Nano ZS instrument (Southborough, MA, USA) was used to measure zeta potentials at 25 °C for all samples. NCL protocol PCC-2 was followed (https://ncl.cancer.gov/resources/assay-cascade-protocols). Samples were prepared at a concentration of 3 mg/mL in 10 mM NaCl. Sample pH was measured before loading into a pre-rinsed folded capillary cell. Measurements were made at both native pH and after adjustment to near neutral pH using 1 N standardized HCl. An applied voltage of 151 V was used. Traces in the figures represent the average of three measurements.

### 4.3. Preparation of NANPs

All sequences are available in the Supporting Information. The strands of DNA for DNA cubes or the DNA templates to produce RNA cubes were purchased from Integrated DNA Technologies (Coralville, IA, USA). RNA cube templates were then PCR-amplified using MyTaq™ Mix from Bioline (London, UK). Purification of the PCR products was done by using a DNA Clean and Concentrator™ kit from Zymo Research (Irvine, CA, USA). T7 RNA polymerase promoters from the PCR products were used to produce RNAs through in vitro run-off transcription with T7 RNA polymerase (80 mM HEPES-KOH (pH 7.5), 2.5 mM spermidine, 50 mM DTT, 25 mM MgCl_2_, 5 mM rNTP). The reaction was incubated at 37 °C for 3.5 h when RQ1 RNase-free DNase (Promega, Madison, WI, USA) was added. Denaturing 8 M urea polyacrylamide gel electrophoresis (PAGE 15%) was used to purify the reactions. RNA bands were visualized under short wavelength UV, cut, and eluted in a crush and soak buffer (300 mM NaCl, 89 mM tris-borate (pH 8.2), 2 mM EDTA) overnight. RNA was precipitated in 2× volume of 100% ethanol for 3 h at −20 °C. 90% ethanol was used to wash the samples after centrifugation at 14,000 RCF for 30 min and twice for 10 min. The supernatant was disposed of and samples were then vacuum-dried and dissolved in double-deionized water (17.8 MΩ*cm). DNA and RNA cubes were each assembled using a one-pot assembly by combining each of the purified monomers at equimolar concentrations in double-deionized and endotoxin-free water. Solutions were then heated to 95 °C and cooled to 45 °C where assembly buffer (89 mM tris-borate (pH 8.2), 2 mM MgCl_2_, 50 mM KCl) was added after 2 min. DNA and RNA cubes were heated at 45 °C for an additional 20 min prior to storage at 4 °C throughout all experiments.

### 4.4. Physicochemical Characterization of NANPs

To analyze the DNA and RNA cube assemblies, 8% non-denaturing native-PAGE (37.5:1) was used in the presence of 89 mM tris-borate (pH 8.2) and 2 mM MgCl_2_. The gels were run for 20 min (Mini-PROTEAN^®^ Tetra system Bio-Rad, Hercules, CA, USA) at 4 °C and 300 V. Gels were washed with double-deionized water and stained for 5 min with ethidium bromide for visualization using a ChemiDoc MP system (Bio-Rad) (Hercules, CA, USA). The resulting single bands for each cubic NANP demonstrate its complete assembly. Atomic force microscopy (AFM) of DNA and RNA cubes was performed on a freshly cleaved 1-(3-aminopropyl)silatrane-modified mica surface using a MultiMode AFM Nanoscope IV system (Bruker Instruments, Santa Barbara, CA, USA) in tapping mode.

### 4.5. Complexing NANPs and Dendrimers 

Gel retardation assays were performed to assess the level at which the positively charged G5-NH_2_ dendrimers could neutralize the negative charge of Alexa 488-labeled DNA duplexes. DNA duplexes and G5-NH_2_ dendrimers were complexed at various N/P ratios and incubated for 30 min at room temperature before being run on a 2% agarose gel for 30 min at 75 V. The gel was imaged using a ChemiDoc MP system (Bio-Rad). To determine the G5-NH_2_ dendrimers’ ability to protect nucleic acids from nuclease degradation, a double-stranded DNA carrying Alexa 488 (5′) and an Iowa Black Quencher (3′) on complementary strands was complexed to the G5-NH_2_ dendrimers at the 1.5 N/1 P ratio. When samples were treated with DNase, digested DNA would result in separation of the fluorophore and quencher and subsequent increase in detection of fluorescence. DNAs were incubated with the G5-NH_2_ dendrimer for 30 min at room temperature. The complexes were then treated with 3 µL of RQ1 RNase-Free DNase (Promega, Madison, WI, USA) and immediately placed into a Bio-Rad C1000 Touch Thermal Cycler with a CFX96 Real-Time System (Hercules, CA, USA) Fluorescence was read every 30 s and the resulting curves were normalized to changes from the baseline fluorescence of the non-treated controls. 

### 4.6. Transmission Electron Microscopy 

After complexation, 5 µL of each sample was dropped onto a carbon-coated 400 mesh Cu/Rh grid (Ted Pella, Redding, CA, USA) and stained with 5 µL of 1% uranyl acetate (Polysciences, Warrington, PA, USA) which was prepared in filtered distilled water. A FEI Talos L120C TEM with a Gatan 4 k × 4 k OneView camera was used to image the grids.

### 4.7. Uptake by Cancer Cell Line MDA-MB-231

To assess uptake of the complexed cubes and dendrimers by a cancer cell line, MDA-MB-231 cells were used. The cells were cultured in DMEM containing 10% heat-inactivated FBS, 100 U/mL penicillin, and 100 μg/mL streptomycin at a density of 40,000 cells per well in a 24-well plate and incubated at 37 °C and 5% CO_2_ in a humidified incubator. After 24 h, the cells were transfected with Alexa 488-labeled cubes and the dendrimer complex at a final concentration of 50 nM of cubes for a period of 24 h. To compare, Alexa 488-labeled cubes were alternatively complexed with L2K (0.5 µL per well) for 30 min at room temperature and transfected into cells at a final concentration of 50 nM of cubes. After the incubation period, the cells were imaged with an EVOS FL Auto Imaging System (Thermo Fisher Scientific) (Carlsbad, CA, USA). The cells were then washed with phosphate buffered saline (PBS) and detached with 0.25% trypsin–EDTA (Thermo Fisher Scientific, Waltham, MA, USA). The detached cells were replenished with media, centrifuged for 5 min at 300× *g*, and the cell pellet was resuspended in PBS. The cells were analyzed with flow cytometry (BD Accuri C6). Cell viability of the MDA-MB-231 cells post-transfection with the cubes and G5-NH_2_ dendrimers was measured using an MTS assay (Cell Titer 96^®^ AQueous One Solution Cell Proliferation Assay from Promega, Madison, WI, USA). MDA-MB-231 cells were plated in a 96-well plate and then transfected with cube–dendrimer complexes at concentrations of 5, 10, 20, 50, and 100 nM. Cell viability was assessed by measuring the relative absorbance of the transfected cells with respect to the non-transfected cells at 490 nm using a Tecan ULTRA microplate reader.

### 4.8. Research Donor Blood

Blood was collected from healthy donor volunteers under NCI-Frederick protocol OH9-C-N046. Each donor was assigned a random number. Blood was collected into vacutainers containing Li-heparin as an anticoagulant and processed to isolate PBMC within 2 h after donation.

### 4.9. In Vitro Cytokine Release 

PBMC isolation and cytokine analysis were performed as described previously [66]. Briefly, NANPs alone, NANPs after complexation with Lipofectamine 2000 or generation 5 amine-terminated PAMAM dendrimers, and positive or negative controls were added to PBMC cultures, and the incubation continued overnight at 37 °C in an incubator with 5% CO_2_. Complexation with Lipofectamine was done using the protocol described by us earlier [66]. For complexation with dendrimers, stocks of NANPs and dendrimers were incubated at room temperature for 30 min, then diluted in the complete cell culture medium (RPMI supplemented with 10% heat-inactivated FBS, 2 mM L-glutamine, 100 U/mL penicillin, and 100 μg/mL streptomycin). The final concentration of NANPs in the culture was 10 nM for all tested conditions (without a carrier, complexed with L2K, and complexed with dendrimers). After the incubation, the culture supernatants were collected and centrifuged at 18,000× *g* for 5 min. The supernatants were then analyzed by multiplex ELISA (Quansys Biosciences, Logan, UT, USA) to determine levels of individual cytokines.

### 4.10. Uptake by Flow Cytometry

PBMCs were either left untreated or incubated in the presence of DNA and RNA cubes alone, complexed with Lipofectamine, or complexed with G5 amine-terminated dendrimers. After 24 h of incubation, the cells were washed to remove the excess particles, reconstituted in the flow cytometry buffer, and analyzed using a Novocyte cytometer (ACEA Biosciences, San Diego, CA, USA). All cubes used for experiments were labeled with Alexa 488 (Integrated DNA technologies, Coralville, IA, USA ). The final particle concentration was 10 nM, the same as was used in the cytokine experiments. The cells were separated according to their forward and side scatter, and the live populations of lymphocytes and monocytes were gated into the green fluorescent channel for the detection of particle uptake. The data analysis was performed using the FCS Express software (DeNovo Software Inc., Pasadena, CA, USA).

### 4.11. Statistical Analysis

Data are presented as the means ± standard deviation (SD) in all studies. For statistical analysis, a one-way analysis of variance (ANOVA) followed by Tukey’s multiple comparison test was performed using GraphPad Prism version 9.0.0 for Windows, GraphPad Software, San Diego, CA, USA, www.graphpad.com. A *p*-value of less than 0.05 was considered statistically significant.

## 5. Conclusions

This study demonstrates that amine-terminated PAMAM dendrimers can be used as delivery carriers for nucleic acid nanoparticles. As a proof of concept, the uptake of two representative NANPs (DNA and RNA cubes) was demonstrated in a human cancer cell line prior to in human PBMCs. Most importantly, the uptake by different immune cells present in the peripheral blood and subsequent cytokine responses differ both quantitatively and qualitatively when NANPs are delivered to the blood cells using different carriers such as L2K and dendrimers.

## Figures and Tables

**Table 1:** Summary of the hydrodynamic diameters for G5-NH_2_ PAMAM dendrimers.

Sample	Z-Avg, nm	PdI	Int-Peak, nm	%Int	Vol-Peak, nm	%Vol
Before filtering	30.4 ± 11.0	0.759 ± 0.188	156.5 ± 11.4	59.7 ± 2.0	5.7 ± 0.1	100 ± 0
After filtering	6.3 ± 0.1	0.143 ± 0.022	7.0 ± 0.1	99.1 ± 1.1	5.1 ± 0.1	100 ± 0

Note: Hydrodynamic size is reported as the intensity-weighted average over all size populations (Z-avg) and the intensity-weighted average (Int-Peak) of a particular range of size populations corresponding to the most prominent peak in the volume distribution plot. PdI is the polydispersity index and a measure of broadness of the size distribution derived from the cumulants analysis. Int-Peak is the intensity-weighted average of the primary peak. %Int is the percentage of the intensity spectra occupied by the primary peak. Vol-Peak is the volume-weighted average over the primary peak. %Vol is the percentage of the volume spectra occupied by the primary peak.

**Table 2:** Summary of the zeta potentials for G5-NH_2_ PAMAM dendrimers.

Sample	pH	ZP, mV
G5-NH_2_	10.5 (native)	+4.6 ± 0.9
G5-NH_2_	7.4	+48.2 ± 3.4

## Data Availability

The data presented in this study are available in this manuscript and in supplementary materials.

## Supplementary Materials

The following are available online, Sequences of constructs used in this project and supporting Figures S1 (Complexation of DNA duplex with G5-NH_2_ dendrimers at various N/P ratios), S2 (Cell viability assay of MDA-MB-231 cells treated with NANPs and G5-NH_2_ dendrimers), and S3 (Cytokine induction by DNA and RNA cubes as a function of the delivery carrier).

## Sample Availability

Samples of the compounds (RNA cubes and DNA cubes) are available from the authors upon request.
