# Supplementary material for: Induction of Cytokines by Nucleic Acid Nanoparticles (NANPs) Depends on the Type of Delivery Carrier
Source: Molecules. 2021 Jan 27;26(3):652. doi: 10.3390/molecules26030652 (PMC7865455; doi:10.3390/molecules26030652)
Supplement: Supplementary file 1 [file molecules-26-00652-s001.pdf]

Article

SUPPLEMENTARY INFORMATION

Induction of Cytokines by Nucleic Acid Nanoparticles (NANPs) Depends on the Type of Delivery Carrier

Yelixza I. Avila [1], Morgan Chandler [1], Edward Cedrone [2], Hannah S. Newton [2], Melina Richardson [1], Jie Xu [2], Jeffrey D. Clogston [2], Neill J. Liptrott [3], Kirill A. Afonin [1,*], and Marina A. Dobrovolskaia [2,*]

1 Nanoscale Science Program, Department of Chemistry, University of North Carolina Charlotte, Charlotte, NC, USA
2 Nanotechnology Characterization Laboratory, Cancer Research Technology Program, Frederick National Laboratory for Cancer Research sponsored by the National Cancer Institute, Frederick, MD, USA
3 Department of Pharmacology and Therapeutics, Institute of Systems, Molecular and Integrative Biology, University of Liverpool, Liverpool, UK
* Correspondence: kafonin@uncc.edu (K.A.A.), marina@mail.nih.gov (M.A.D.)

Sequences used in this work. One strand per NANP was modified with an Alexa 488 label to assess uptake by fluoresence.

Six-stranded DNA cube:

5′- GGCAACTTTGATCCCTCGGTTTAGCGCCGGCCTTTTCTCCCACACTTTCACG

5′- GGGAAATTTCGTGGTAGGTTTTGTTGCCCGTGTTTCTACGATTACTTTGGTC

5′- GGACATTTTCGAGACAGCATTTTTTCCCGACCTTTGCGGATTGTATTTTAGG

5′- GGCGCTTTTGACCTTCTGCTTTATGTCCCCTATTTCTTAATGACTTTTGGCC

5′- GGGAGATTTAGTCATTAAGTTTTACAATCCGCTTTGTAATCGTAGTTTGTGT

5′- GGGATCTTTACCTACCACGTTTTGCTGTCTCGTTTGCAGAAGGTCTTTCCGA

Fluorescently labeled DNA cube strand:

5′- GGCGCTTTTGACCTTCTGCTTTATGTCCCCTATTTCTTAATGACTTTTGGCC/**Alexa 488**/

Six-stranded RNA cube:

5′- GGCAACUUUGAUCCCUCGGUUUAGCGCCGGCCUUUUCUCCCACACUUUCACG

5′- GGGAAAUUUCGUGGUAGGUUUUGUUGCCCGUGUUUCUACGAUUACUUUGGUC

5′- GGACAUUUUCGAGACAGCAUUUUUUCCCGACCUUUGCGGAUUGUAUUUUAGG

5′- GGCGCUUUUGACCUUCUGCUUUAUGUCCCCUAUUUCUUAAUGACUUUUGGCC

5′- GGGAGAUUUAGUCAUUAAGUUUUACAAUCCGCUUUGUAAUCGUAGUUUGUGU

5′- GGGAUCUUUACCUACCACGUUUUGCUGUCUCGUUUGCAGAAGGUCUUUCCGA

Fluorescently labeled RNA cube strand:

5′-
GGCGCUUUUGACCUUCUGCUUUAUGUCCCCUAUUUCUUAAUGACUUUUGGCC/**Alex a 488**/

DNA duplex used in DNase protection assays:

5′- /5IABkFQ/TGACCCTGAAGTTCATCTGCACCACCGGTCACGGTCTCC

5′- GGAGACCGTGACCGGTGGTGCAGATGAACTTCAGGGTCATT/**Alexa 488**/

DNA duplex used in complexation to determine N/P ratio:

5′- TGACCCTGAAGTTCATCTGCACCACCGGTCACGGTCTCC

5′- GGAGACCGTGACCGGTGGTGCAGATGAACTTCAGGGTCATT/3AlexF488N/

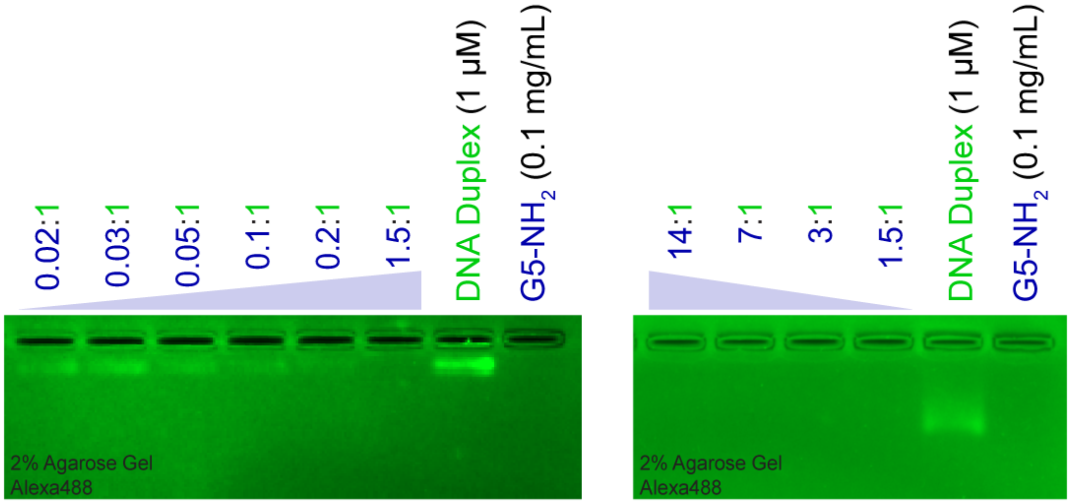

Figure S1. Complexation of DNA duplex with G5-NH$_2$ dendrimers at various N/P ratios. DNA duplexes labeled with Alexa 488 (labeled in green font) were combined with G5-NH2$_2$ dendrimers (labeled in blue font) at different negative charge (N) to positive charge (P) ratios as shown above. The samples were then analyzed by agarose gel electrophoresis.

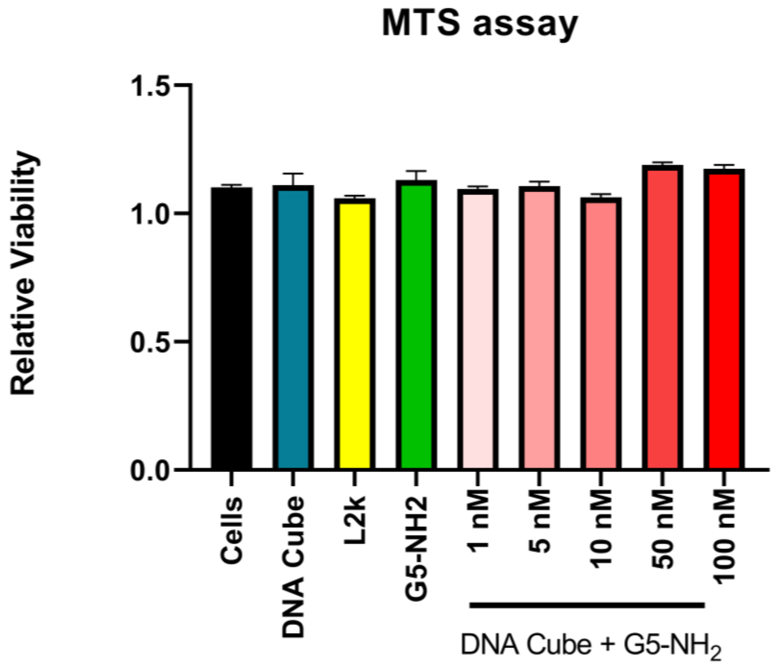

Figure S2. Cell viability assay of MDA-MB-231 cells treated with NANPs and G5-NH_2 dendrimers. Viability of MDA-MB-231 cells after being exposed to DNA cube G5-NH2 dendrimer complexes which was evaluated at 72 hours. All samples remained above an 80% viability.

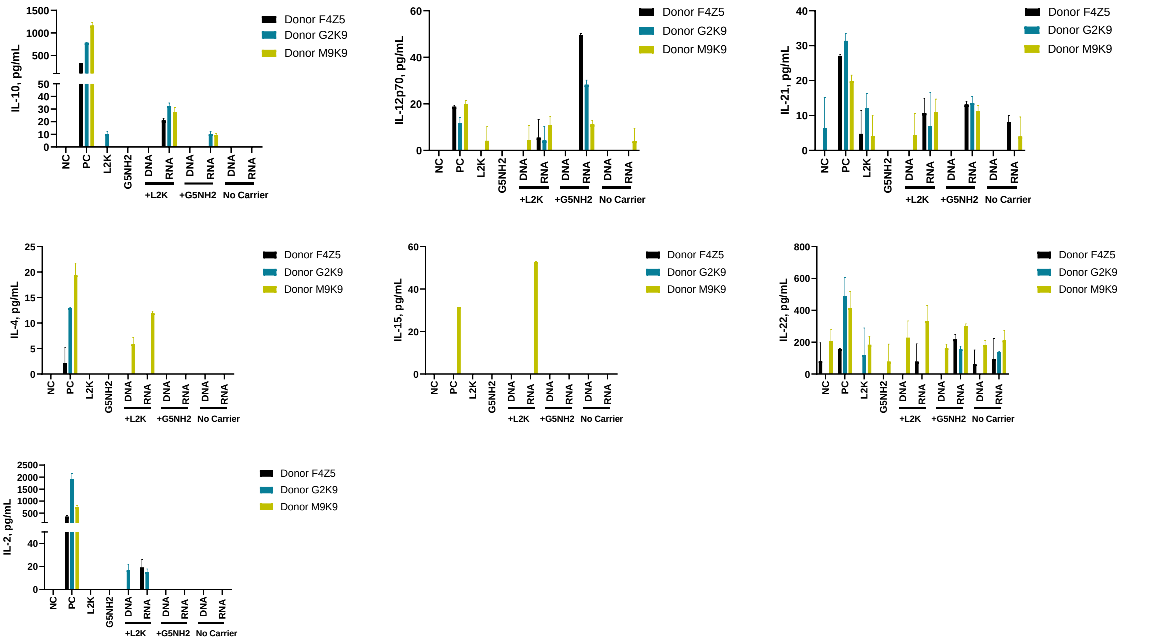

Figure S3. Cytokine induction by DNA and RNA cubes as a function of the delivery carrier. PBMC from three healthy human donor volunteers (F4Z5, G2K9, and M9K9) were treated with negative control (NC), positive control (PC), DNA cubes, or RNA cubes for 24 hours. Prior to the addition to PBMC cultures, DNA cubes and RNA cubes were complexed with lipofectamine 2000 (L2K), G5 amine-terminated PAMAM dendrimers (G5-NH_2) or used without complexation (no carrier). Culture supernatants were analyzed for the presence of cytokines, chemokines, and interferons using multiplex ELISA as described in Materials and Methods. The data for other cytokines grouped based on their function (i.e., type I and type III interferons, danger signals and cytokines commonly associated with trauma and cytokine storm, type II interferon and type II interferon inducible protein, and chemokines are presented on Figure 5. Each bar shows the mean response and standard deviation ($N = 2$).
